# A radiological severity scale to measure the impact of Charcot’s Neuroarthropathy: an observational study

**DOI:** 10.1186/s13047-020-0375-5

**Published:** 2020-02-11

**Authors:** Shan Bergin, Parm Naidoo, Cylie M. Williams

**Affiliations:** 1 Monash Health, Department of Podiatry, David Street, Dandenong, VIC 3175 Australia; 2 Monash Health, Monash Imaging, David Street, Dandenong, VIC 3175 Australia; 3 Monash Health, Allied Health Research Unit, Warrigal Rd, Cheltenham, VIC 3192 Australia; 4 Department of Physiotherapy, Monash University, McMahon’s Road, Frankston, VIC 3199 Australia

**Keywords:** Charcot’s neuroarthropathy, Diabetic neuropathy, Diabetic foot, Radiological imaging, Severity scale

## Abstract

**Background:**

Charcot’s Neuroarthropathy (Charcot foot) is a debilitating and destructive disorder resulting from neurological changes in the foot. Whilst the majority of cases are painless, as a result of disruption to sensory function, a common outcome is severe deformity that impacts considerably on foot function. The purpose of this study was to develop and validate a radiological severity scale to quantify resultant damage from acute mid foot Charcot’s. This in turn can be used to evaluate clinical outcomes related to different degrees of offloading.

**Methods:**

A four round Delphi process was used to develop five tool items. Level of consensus and agreement was set at 80%. Inter-rater and intra-rater reliability was evaluated using 3 raters and 24 plain x-rays of chronic mid-foot Charcot’s. Strength of agreement of individual items and overall scores was calculated using weighted Kappa coefficients (S.E). Cronbach’s α was used to determine internal consistency. Floor (> 15% score 0) and ceiling (> 15% score 11) effects were examined at each time point. Spearman’s correlation coefficient was used to assess construct validity using Mobility and Usual Activity scores taken from the EQ-5D-5 L.

**Results:**

Twenty two patients participated. The five item severity scale demonstrated a Cronbach’s α of 0.91. Intra-rater Kappa coefficients (SE) for total scores ranged from 0.84 (0.20) to 0.86 (0.20). Inter rater coefficients (SE) ranged from 0.72 (0.14) to 0.83 (0.14). Distribution was normal and no floor or ceiling effects were identified.

**Conclusion/interpretation:**

This study suggests it may be possible to quantify resultant damage from mid foot Charcot’s. Given the physical and emotional impacts from long periods of complete immobilisation defining a minimum standard would be an important development in the management of Charcot foot.

## Introduction

Charcot’s Neuroarthropathy (Charcot foot) is a debilitating disorder resulting from neurological changes in the foot [1]. Known to affect several patient populations, it predominantly presents in those with diabetes [2]. Whilst the majority of cases are painless, as a result of disruption to sensory function, a common outcome is severe deformity that impacts considerably on foot function. The end result for many people who develop Charcot foot is ulceration and infection over areas of increased pressure thereby increasing the risk of lower limb amputation [3]. A 2011 paper by Rogers et al. describes Charcot foot as an inflammatory disorder that alters bone density [4]. This in turn acts as a pre cursor to bone fracture or fragmentation, and joint subluxation or dislocation that often goes unheeded until such time as a major deformity is evident. During this process, bone shape commonly changes in the midfoot. Charcot foot is self-limiting and little can be done to halt the acute inflammatory process once it begins with the duration of the acute phase varying widely between affected patients.

The consensus regarding management of Charcot foot has been the use of immobilisation for maintaining foot structure and therefore function [4, 5]. During this time the foot itself is immobilised in a non-removable cast, but the degree to which clinicians recommend patient immobilisation with gait aids or a wheelchair is highly variable. Available recommendations suggest anything from confinement to a wheelchair with strictly no weight bearing on the affected limb, to fashioning the cast to allow weight bearing so patients can mobilise to perform activities of daily living [6]. Both options have potential to result in adverse effects including generalised osteoporosis and deconditioning with use of a wheelchair and likelihood of further structural damage with any degree of weight bearing [7]. Given the acute phase of Charcot foot can last several months, confinement to a wheelchair would have significant effects on quality of life which in turn can reduce overall patient adherence. What is unclear is whether more or less patient immobilisation results in the same or different clinical outcomes.

Several staging scales have been developed aiming to describe the course of the Charcot foot over time [8]. The first and most widely recognised is the Eichenholtz Classification System that initially presented as a 3 -point scale, used to describe what is occurring physiologically, but was later modified to include a fourth stage [9]. Stage 0 indicates the presence of oedema with normal radiological images. Stages 1–3 refer to the progression through acute, sub-acute and chronic phases. At each stage the classification system can also be used to guide staged treatment during the natural progression of the disease. What this and other classification systems do not do, is allow for quantification of the damage caused by the acute inflammatory process.

The aim of this study is to determine whether a valid and reliable radiological severity scale can be developed. Such a scale has the future potential for use in broader research to identify which treatment regimens provide the best clinical outcome whilst imposing the least amount of physical and emotional distress.

## Methods

A two stage research design was implemented. The first stage consisted of, a Delphi panel process for item development and wording refinement. The second stage consisted of a validation cohort study to test the scale. The Monash Health Human Research Ethics Committee approved this research (12263 L).

### Stage 1 - Delphi panel process

An online Delphi panel was undertaken to determine expert agreement and was facilitated by a member of the research team (CW). The Delphi technique is a valid method to determine consensus. It involves sequential questionnaires answered by a panel of participants with relevant expertise to gain consensus [10]. The Delphi panel recruited radiologists (*n* = 2) and podiatrists (*n* = 3) who had recognised experience in the diagnosis and management of Charcot foot in Australia and New Zealand. These invited health professionals were identified based on their clinical experience with Charcot foot and/or their contribution to evidence within the field of Charcot foot diagnosis and/or management. Each panel member held senior clinical positions within their respective organisations, and had more than 10 years experience with this patient cohort. The surveys in each round were implemented using Survey Monkey [11]. Participants were considered enrolled once they supplied written informed consent. Panel members were geographically diverse and were asked to keep their participation confidential and all intra-panel communication was anonymous and non-discipline specific. The initial round was used to gain an understanding of expert perception of measurement and structural change of the foot affected by Charcot’s, responses were then collated and analysed for consensus [12]. Any responses not reaching consensus were returned to panel for agreement in subsequent rounds. Level of consensus and agreement were set at 80% due to the small number of panel members.

In round one, Delphi panel members were asked to list radiographic indicators of Charcot foot on plain film and MRI, potential importance weighting of indicators, plain film clinical features affecting foot function, commonly used radiological techniques for diagnosing Charcot foot, opinion on quantification of damage with Charcot foot. Subsequent rounds utilised statements generated from Round 1 to determine agreement of the statement on a 5 point Likert scale where 1 was Strongly disagree, 2 was Disagree, 3 was Neutral, 4 was Agree and 5 was Strongly Agree. In the subsequent rounds, statements were considered accepted if 80% or more participants indicated they ‘Agreed’ or ‘Strongly Agreed’ with the statement. Statements not reaching 40% agreement were excluded from the subsequent rounds. Statements receiving 40–60% agreement were reviewed in subsequent rounds to ensure adequate panel consideration. Statements were excluded if agreement had not been achieved within two rounds. The Delphi would be concluded when the response rate dropped below 80% or when round 4 was completed irrespective of agreement. Each round was open for 3 weeks and there was between 1 and 6 weeks between feedback to participants and subsequent round.

### Stage 2 – Validation with the study population

Cohort participants included a convenience sample of 22 participants (24 feet) from Monash Health Podiatry Department who were diagnosed with midfoot Charcot’s through history of diabetes mellitus, peripheral neuropathy, adequate vascularity and a difference in temperature to the unaffected limb, with or without radiological changes. These patients had subsequently undergone treatment for midfoot Charcot’s. For inclusion in the study, patients were required to have no active Charcot foot, be willing to have their radiological plain films reviewed by the study team and complete a quality of life measure (EQ-5 L-5D). Consenting participants were contacted via phone for permission to send the paperwork which they then returned via a stamped, addressed envelope to the study team.

The EQ-5D-5 L is a validated quality life measure designed primarily for ‘self completion’. Developed by EuroQOL the EQ-5D-5 L measures five health domains: Mobility, Self-Care, Usual Activities, Pain/Discomfort, and Anxiety/Depression [13]. Patients select the degree of ‘problems’ they experience in each domain and also rate their overall health at a single point in time using a 0–100 visual scale.

### Data analysis

All data were analysed using Stata 13 [14]. In Stage 1, the scale was developed through the Delphi process. In stage 2, the scale was validated with the research team members.. Rater 1 (SB) had 23 years of clinical experience managing and diagnosing Charcot foot as a podiatrist and is referred to throughout the study as the Expert Podiatrist; Rater 2 (PN) had 20 years of radiological experience reporting radiological outcomes of Charcot foot and is referred to throughout the study as the Radiologist; Rater 3 (CW) has 20 years of clinical experience as a podiatrist utilising plain films during treatment of general podiatry patients (excluding patients with Charcot foot) and is referred to throughout the study as Novice Podiatrist. The two Podiatrists who participated in this phase of the study did not participate as members of the ‘expert’ Delphi Panel.

The raters reviewed each question together and trialled the scale with three plain films not included within the study to ensure consistency in interpretation of any profession specific nuances of the tool. A list of 24 plain film series were compiled and the raters undertook scoring these individually with the tool. Scores were entered into an online survey to reduce recall bias. The raters completed a second scoring of the same 24 film series within the subsequent week.

Weighted kappa coefficients (S.E) were calculated to assess the strength of agreement of inter and intra-rater reliability of the individual questions and total score. A kappa greater than 0.80 was classed as very good agreement, 0.61–0.80, good agreement, 0.41–0.60 as moderate agreement, 0.21–0.40 as fair agreement and < 0.20 as poor agreement [15, 16]. Crohnbach’s α was calculated to assess the internal consistency of the scale.

The scales responsiveness was examined for floor and ceiling effects at each time point. Floor and ceiling effects were defined as over 15% of respondents achieving a score of 0 (floor) or 11(ceiling) [17].

The construct validity was determined by analysis of the convergent and discriminant validity as there is no gold standard measure of osseous destruction related to Charcot foot. Convergent validity was measured by determining the correlation with a measure of related construct and discriminant validity by a low correlation with an unrelated construct. The convergent validity and divergent validity was assessed using the Spearman’s correlation coefficient of the total score at each time with the Mobility and Usual Activities (convergent) scores of the EQ-5 L-5D and the Anxiety/Depression (divergent) scores of the EQ-5 L-5D.

A minimum sample size of 19 was calculated to provide 80% power of detecting a correlation of 0.6 with a two-tailed alpha = 0.05 for the intra-rater reliability analysis [15].

## Results

### Delphi panel consensus

There were five experts recruited to participate in the Delphi process. Two radiologists, and three Podiatrists with recognised expertise in diagnosis and/or management of Charcot foot.

During Round 1 experts were also asked to rank plain film clinical features of Charcot foot in order of perceived importance in terms of foot function affect with both multi-choice and open ended questions. Comments were collated and common themes identified and modified into statements by discussion and agreement with authors. There were 23 statements initially generated in Round 1 with nine achieving consensus and these statements were returned to the panel within Round 2 of the process.

### Agreement

**Fig. 1:**
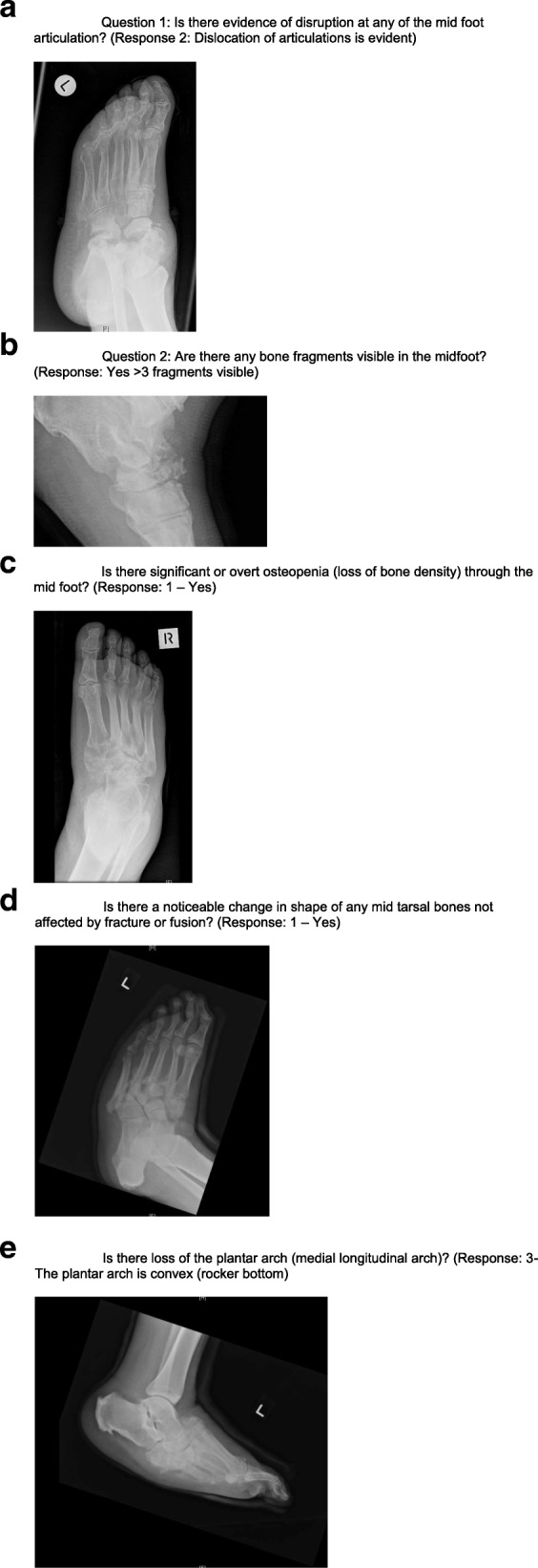
**a** Question 1: Is there evidence of disruption at any of the mid foot articulation? (Response 2: Dislocation of articulations is evident). **b** Question 2: Are there any bone fragments visible in the midfoot? (Response: Yes > 3 fragments visible). **c** Is there significant or overt osteopenia (loss of bone density) through the mid foot? (Response: 1 – Yes). **d** Is there a noticeable change in shape of any mid tarsal bones not affected by fracture or fusion? (Response: 1 – Yes). **e** Is there loss of the plantar arch (medial longitudinal arch)? (Response: 3- The plantar arch is convex (rocker bottom)

During Round 2, the participants reviewed the 15 statements where no consensus was achieved and participant levels of agreement were measured. There were two statements achieving 80% or more agreement, three statements with between 60 and 80% agreement and ten statements achieving less than 40% agreement. The third round incorporated the three statements and all consensus and agreement statements were placed into a proposed multi-choice tool format for the Delphi panel to consider. This tool received 80% (*n* = 4) agreement for the format and wording representative of the panel’s answers specific to components required to consider the radiological measurement of Charcot foot severity. The remaining respondent agreed to the tool content but proposed terminology/grammar clarification only and these changes were made within the final draft. All participants received a final copy of the tool and the final questions with ranked importance (Table 1). Figure 1a-e are images indicating the highest score possible for each question.

**Table 1:** Questions included in the Charcot Radiological Staging Scale

	Text	Score/response
Question 1	Is there evidence of disruption at any of the mid foot articulation? (Do not take fusion into consideration and default answer to the highest level of disruption)	0 - No 1 - Subluxation of articulations is evident 2 - Dislocation of articulations is evident (Fig. 1a)
Question 2	Are there any bone fragments visible in the midfoot?	0 - No 1 - Yes 1–3 fragments visible 2 - Yes > 3 fragments visible (Fig. 1b)
Question 3	Is there significant or overt osteopenia (loss of bone density) through the mid foot?	0 - No 1 – Yes (Fig. 1c)
Question 4	Is there a noticeable change in shape of any mid tarsal bones not affected by fracture or fusion?	0 - No 1 – Yes (Fig. 1d)
Question 5	Is there loss of the plantar arch (medial longitudinal arch)?	0- No the plantar arch is intact 1- Yes there is some lowering of the plantar arch 2- Yes the plantar arch has been lost 3- The plantar arch is convex (rocker bottom) (Fig. 1e)

The tool was then utilised to determine its psychometric properties with the 22 participants and initial analysis of the internal consistency was calculated with Cronbach’s α of 0.59. Person items were visually examined for disparity between scores and the removal of one person from the scale resulted in a Cronbach’s alpha of 0.91. This person’s score was found to have a total score difference of 4 points between the two time-points when scored by the novice rater and at least 3 points between the novice rater scores and the experienced podiatrist and radiologist scores. The experienced podiatrist and radiologist all have the same scores for this person’s score. No other person’s scores had such variation between time-points or raters. All subsequent analyses were undertaken with the 22 participant scores only.

The 22 participants (24 feet) were diagnosed with Charcot foot through a specialist high risk foot clinic. There were 11 left foot Charcot’s, 9 right foot Charcot’s and 2 participants with bilateral Charcot’s. Participants had mean (SD) age of 62.3 (9.7) years and 56% (*n* = 14) were male. Table 2 displays the outcomes of the analysis and the median total scores, the intra and inter-rater reliability of the questions and the total scores for each of the raters and rater pairs.

**Table 2:** Intra and Inter-rater reliability of Questions 1 to 5 on the Charcot Radiological Staging Scale and the total score

Measure	Rater	Q1 *Kappa* (SE^a^)	Q2 *Kappa* (SE^a^)	Q3 *Kappa* (SE^a^)	Q4 *Kappa* (SE^a^)	Q5 *Kappa* (SE^a^)	Median total score (IQR^b^)	Total Score *Kappa* (SE^a^)
Intra-rater	Novice Podiatrist	0.84 (0.19)	0.86 (0.20)	0.44 (0.20)	0.78 (0.20)	0.73 (0.20)	5 (3–6)	0.84 (0.20)
	Expert Podiatrist	0.49 (0.20)	0.80 (0.20)	0.58 (0.19)	0.65 (0.19)	0.86 (0.20)	5 (4–6)	0.86 (0.20)
	Radiologist	0.82 (0.19)	0.87 (0.20)	0.67 (0.20)	0.58 (0.20)	0.84 (0.20)	4.5 (3–6)	0.86 (0.20)
Inter-rater	Novice Podiatrist/ Expert Podiatrist	0.61 (0.13)	0.78 (0.14)	0.32 (0.14)	0.46 (0.14)	0.79 (0.14)		0.83 (0.14)
	Novice Podiatrist/ Radiologist	0.75 (0.14)	0.77 (0.14)	0.31 (0.14)	0.09 (0.08)	0.70 (0.14)		0.72 (0.14)
	Expert Podiatrist/Radiologist	0.58 (0.12)	0.65 (0.14)	0.36 (0.14)	0.10 (0.06)	0.74 (0.14)		0.74 (0.14)

^a^*SE* Standard Error, ^b^*IQR* Interquartile Range

**Fig. 2:**
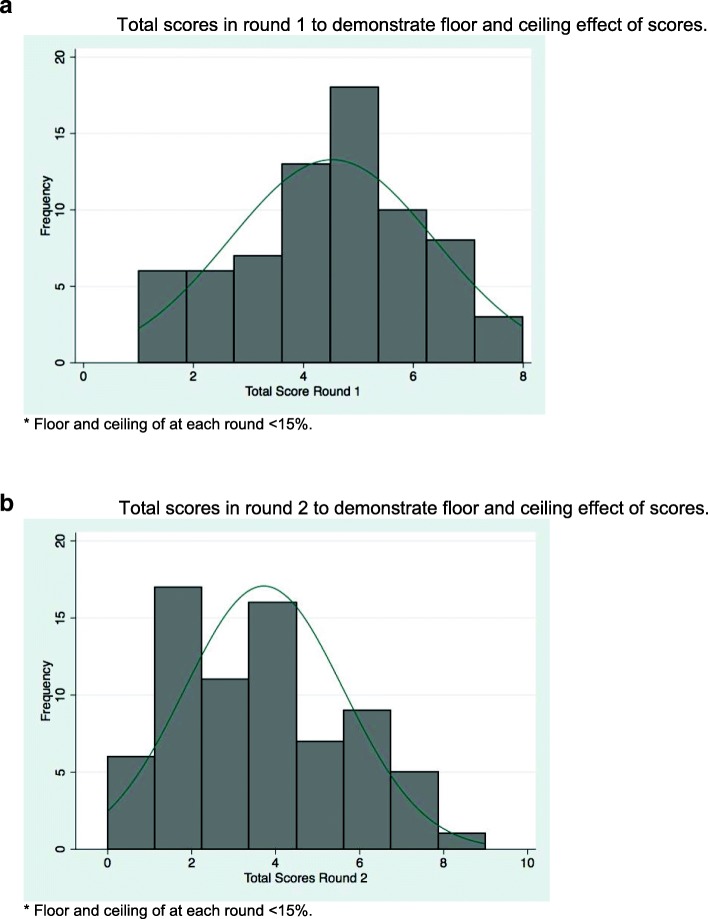
**a** Total scores in round 1 to demonstrate floor and ceiling effect of scores. * Floor and ceiling of at each round < 15%. **b** Total scores in round 2 to demonstrate floor and ceiling effect of scores. * Floor and ceiling of at each round < 15%

The floor and ceiling effects were determined and Fig. 2 shows the differences between the distribution of total scores at each time point. There was normal distribution and no floor or ceiling effect at either Round 1 or Round 2.

**Fig. 3:**
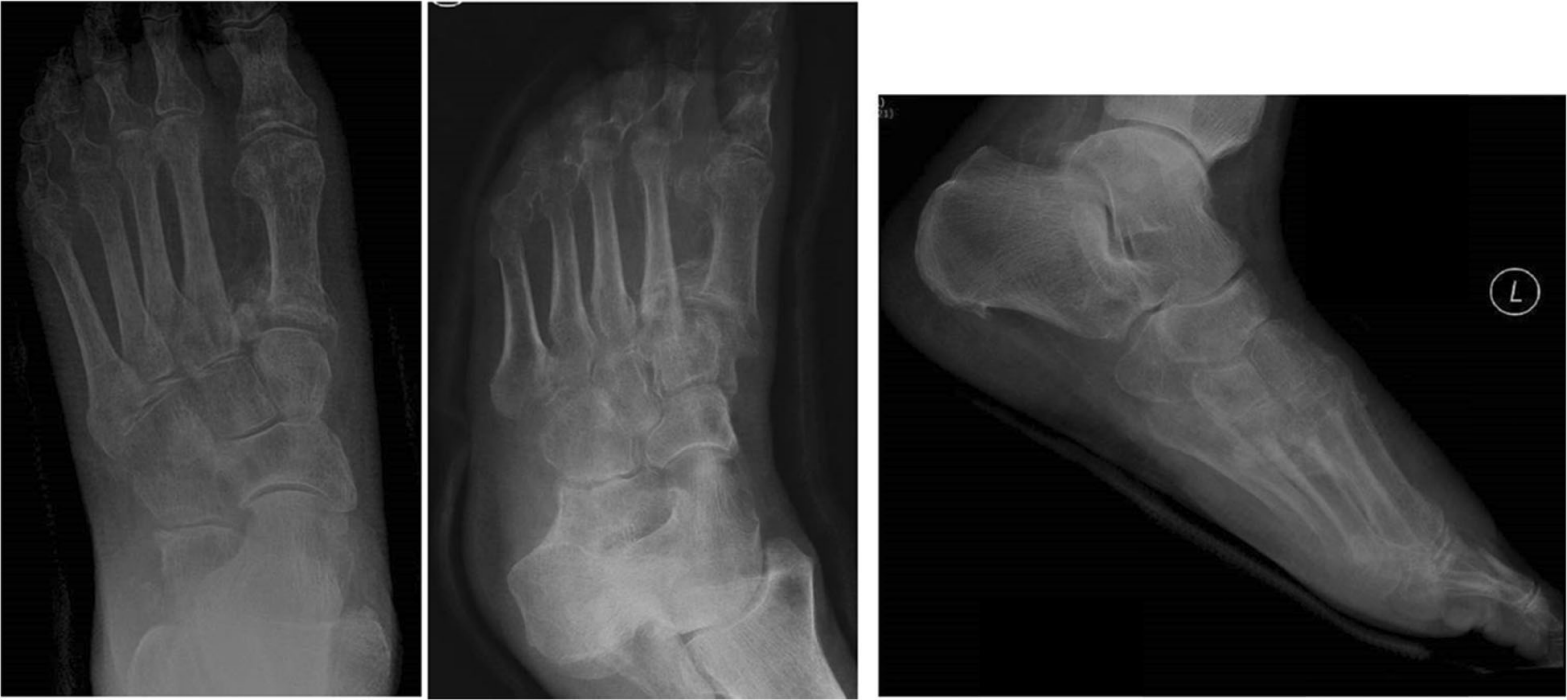
Radiograph of participant who scored a mean of 7.2 out of 11 on the severity scale and rated their quality of life 95/100

The quality of life results were used as a pseudo measure of functional ability and the overall impact of the foot deformity. Figure 3 displays a radiograph of a participant who scored a mean (SD) score of 7.2 (0.4) from a possible 11, from the six times their x-ray was viewed during reliability testing. However, the patient also rated their health as 95 on a scale of 0–100 where 100 is the best possible health and scored themselves as a 2 in the mobility domain of the EQ-5 L-5D, “*I have slight problems walking around*”. When examining the validity of the tool this was reflected in many of the scores.

There was no evidence of convergent validity for the total score, a low and non-significant correlation with the ‘Mobility’ (*r* = 0.19, *p* = 0.38) and ‘Usual Activities’ (*r* = 0.22, *p* = 0.30) scores of the EQ-5 L-5D. There was evidence of divergent validity for the total score with a low and non-significant correlation with the (*r* = − 0.17, *p* = 0.50).

## Discussion

This study shows promise of a tool to quantify the radiological changes that the foot undergoes during the development of mid foot Charcot’s. Recent research into Charcot foot has largely focussed on attempting identification of the risk factors for Charcot foot development, understanding Charcot foot pathophysiology and defining incidence and prevalence [4, 18]. Studies have also explored the impact of Charcot foot on functional ability and quality of life [19, 20]. Yet there is minimal research providing an evidence base for the treatment options commonly provided to individuals who experience Charcot foot.

The treatment approach to Charcot foot has remained largely unchanged since the 1990’s [21]. Whilst the need to minimise the degree of force affecting the foot during the acute phase is not in dispute, how this is achieved and the degree to which functional forces need to be reduced is not clear. The first step in attempting to standardise treatment protocols that are evidence based, clinically effective and patient centred is to develop a reliable method of measuring treatment outcomes. The development of this radiological severity scale attempts to provide the means to facilitate further investigation in to the effect of different treatment regimens on structural damage from mid foot Charcot’s.

To develop and validate such a tool is not without difficulty. While the raters were undertaking standardisation of terms prior to the individual reviews there were a number of key challenges they observed. Reading of plain films is often subjective and within this study, the biggest challenge was that there is no standardised measure of bone density and there is often variability in bone shape seen in individuals. These two items were the ones that had the poorest inter-rater reliability even though reliability was still within the acceptable range. It is possible that with training using x-ray examples that the reliability may increase between raters. The other questions had greater reliability potentially as there was less variability in the response ie: consistent definition of subluxation or dislocation and how it is viewed on the plain film (Qn 1) and fragments could easily be counted (Qn 2). An additional challenge to the development of a tool to quantify change is that there is no correlation between the radiological appearance of a typical Charcot foot and the corresponding EQ-5 L-5D participant score.

This tool shows promise for future research despite a number of key limitations. These related to the transferability of results at this stage of the development of the tool. The author group spent significant time discussing each item within the tool to ensure each understood the measures. This discussion was in person and training in tool use should be considered prior to further use. This tool was developed with a classical test theory approach and with a small sample size. Larger sample sizes would allow additional statistical analysis such as item response theory additional testing of internal consistency and performance with a wider range of presentations of Charcot foot. Recognition that removal of one person’s plain films from the analysis substantially influenced the internal consistency in a small sample size leads to cautious interpretation of this tool. While the tool had overall acceptable rater reliability, two of the five questions had less than acceptable reliability. This may be mitigated with additional training prior to scoring, however, is presently a limitation of the tool scoring. The next stage of development should look at use of the tool with different skilled raters in a larger cohort of participants. Additionally, this tool should be used with and without a Charcot foot diagnosis to ensure it identifies the key features. This will allow further exploration of the tool and its ability to quantify radiological changes. The ability to quantify radiological changes will enable much needed research into the most effective method to reduce the impact of mid foot Charcot’s.

## Conclusion

On a background of chronic disease, disorders such as Charcot foot add another level of complexity for patients already struggling to comply with treatment recommendations. Wherever possible clinicians should attempt to implement treatment options that are evidence based and have minimum impact on overall quality of life. Tools such as the Charcot’s Radiological Severity Scale have potential to inform minimum standards for off-loading this patient group.

## Data Availability

The data that support the findings of this study are available from the corresponding author in anonymised form upon reasonable request.
